# Mangrove diversity is more than fringe deep

**DOI:** 10.1038/s41598-022-05847-y

**Published:** 2022-02-01

**Authors:** Steven W. J. Canty, John Paul Kennedy, Graeme Fox, Kenan Matterson, Vanessa L. González, Mayra L. Núñez-Vallecillo, Richard F. Preziosi, Jennifer K. Rowntree

**Affiliations:** 1 Smithsonian Marine Station, 701 Seaway Drive, Fort Pierce, FL 34949 USA; 2 Working Land and Seascapes, Smithsonian Institution, Washington, DC 20013 USA; 3 Department of Natural Sciences, Ecology and Environment Research Centre, Manchester Metropolitan University, Manchester, M1 5GD UK; 4 National Museum of Natural History, 10th Street and Constitution Avenue,, Washington, DC 20560 USA; 5 Global Genome Initiative, National Museum of Natural History, 10th Street and Constitution Avenue, Washington, DC 20560 USA; 6 The Coral Reef Alliance, Mesoamerican Region, 1330 Broadway, Suite 600, Oakland, CA 94612 USA; 7 School of Biological and Marine Sciences, University of Plymouth, Plymouth, PL4 8AA UK

**Keywords:** Ecological genetics, Forest ecology

## Abstract

Mangroves form coastal tropical forests in the intertidal zone and are an important component of shoreline protection. In comparison to other tropical forests, mangrove stands are thought to have relatively low genetic diversity with population genetic structure gradually increasing with distance along a coastline. We conducted genetic analyses of mangrove forests across a range of spatial scales; within a 400 m^2^ parcel comprising 181 *Rhizophora mangle* (red mangrove) trees, and across four sites ranging from 6–115 km apart in Honduras. In total, we successfully genotyped 269 *R. mangle* trees, using a panel of 677 SNPs developed with 2b-RAD methodology. Within the 400 m^2^ parcel, we found two distinct clusters with high levels of genetic differentiation (F_*ST*_ = 0.355), corresponding to trees primarily located on the seaward fringe and trees growing deeper into the forest. In contrast, there was limited genetic differentiation (F_*ST*_ = 0.027–0.105) across the sites at a larger scale, which had been predominantly sampled along the seaward fringe. Within the 400 m^2^ parcel, the cluster closest to the seaward fringe exhibited low genetic differentiation (F_*ST*_ = 0.014–0.043) with the other Honduran sites, but the cluster further into the forest was highly differentiated from them (F_*ST*_ = 0.326–0.414). These findings contradict the perception that genetic structure within mangroves forests occurs mainly along a coastline and highlights that there is greater genetic structure at fine spatial scales.

## Introduction

Mangroves are a group of highly specialized plants that have adapted to live in the harsh conditions of the intertidal zone along tropical and subtropical coastlines^1^. Mangroves provide critical ecosystem services such as fisheries^2^, protection from hurricanes and storms^3,4^, flood events^5^, and climate change mitigation, through carbon sequestration^6^. Despite their importance, mangrove cover has significantly declined since the 1960’s^7^ and these systems have become more fragmented^8^. There was a real concern that if deforestation rates continued at the historical rates of the 1980’s and 1990’s we faced a world without mangroves^9^. However, over the past decades, mangrove deforestation rates have significantly slowed, and there is cause for cautious optimism^10,11^.

There are calls for greater protection of mangroves, with aims of no net loss and to increase mangrove cover through restoration over the coming years, and such efforts will start to be expanded as we enter the United Nations Decade of Ecosystem Restoration. Maintaining genetic diversity of extant and restored mangrove forests provides the opportunity for natural selection to occur in response to changing environmental conditions^12^. Mangroves are considered to have limited genetic structure at fine-spatial scales (≤ 200 m) due to life history traits, which can facilitate high self-pollination rates^13,14^, and high rates of self-recruitment, which are common in established mangrove stands, particularly in species that produce large propagules, such as *Rhizophora* spp.^15,16^. Additionally, habitat discontinuities can form strong barriers to gene flow^17^.

Population genetics studies have been used to assess genetic connectivity of mangrove populations at estuary^18^, seascape^19^, regional^20^ and biogeographic scales^21^, with low to moderate genetic diversity observed. There has been less focus at small spatial scales (< 200 m), due to the presumed limited genetic diversity at the forest scale, but see Iuit et al.^22^, Ngeve et al.^23^ and Triest et al.^24^. However, fine-scale genetic analyses of other habitat forming foundational species, the scleractinian coral *Acropora cervicornis*^25^ and three species of bulrush, *Bolboschoenus maritimus*, *Schoenoplectus acutus* and *S. americanus*^26^ revealed higher levels of genetic diversity than previously expected, which has major implications for spatial management and restoration practices of these species. Understanding mangrove genetic structure, at various spatial scales, is therefore critical in informing management and restoration strategies, and population genetics should be a key component of management and restoration frameworks (see Mijangos et al.^27^).

Here we conduct population genetic analyses of *Rhizophora mangle* (red mangrove), an iconic mangrove species found along the Atlantic shores of Latin American, the Caribbean and African countries, at fine (≤ 20 m) and larger spatial scales (6–115 km). An isolated *R. mangle* forest was selected to test the hypothesis of limited genetic differentiation at fine, within forests, spatial scales. We also sampled from the seaward forest fringe of three other mangrove stands located along the Honduran north shore to place the fine scale samples in a broader context. We observed limited genetic structure across the seascape for populations of mangroves sampled along the seaward fringe of a forest. However, significant genetic structure was observed at fine spatial scales, with higher levels of genetic differentiation within the forest than expected. Our findings have important implications for the management and restoration of mangrove forests.

## Results

### Fine-scale genetic structure

**Figure 1:**
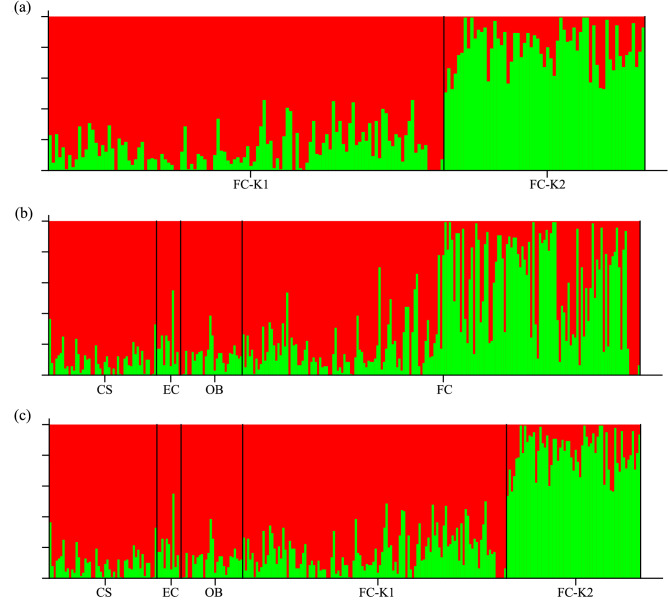
Structure outputs of Rhizophora mangle stands in Honduras. **(a)** Four principal sampling sites, *CS* Cuero y Salado, *EC* Elijah canal, *OB* Oyster bed lagoon, and *FC* Fort cay; **(b)** 400 m^2^ parcel of forest at Fort Cay; and **(c)** Fort Cay divided into two sites, FC-K1 and FC-K2.

**Figure 2:**
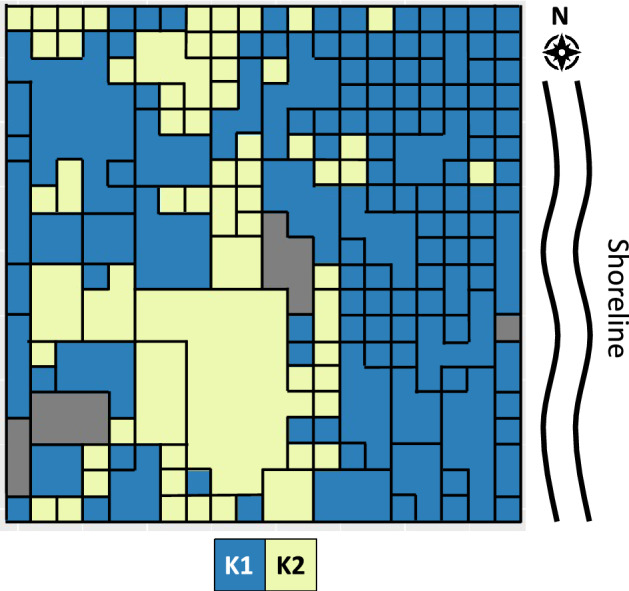
Geographic distribution of individual Rhizophora mangle trees within the 400 m^2^ parcel of forest on Fort Cay as assigned by K-means clustering analysis. Black outlines demark individual trees and gray squares represents no tree present (A13) or samples that did not sequence (I10, Q16, T17).

STRUCTURE analysis identified the potential of two clusters within the parcel of forest located on Fort Cay (Fig. 1a). This was corroborated by K-means clustering analyses which assigned individual trees to one of two clusters (Fig. 2). Trees of varying size, potentially related to age, were associated with each of the two clusters identified, with individuals assigned to cluster K1 primarily associated with the seaward fringe of the parcel, and those of K2 generally located towards the interior of the parcel (Fig. 2). Pairwise F_*ST*_ analyses of the two clusters revealed significant genetic differentiation (F_*ST*_ = 0.355) within the 400m^2^ parcel (Table 1a).

**Table 1:** Pairwise F_*ST*_ of Honduran *Rhizophora mangle* stands.

(a) Fort cay only		Fort cay K1	Fort cay K2			
F_ST_	Fort cay K1 (n = 120)	–	**0.0001**			
	Fort cay K2 (n = 61)	0.355	–			

**(**b) All four sampling sites		Cuero y Salado	Elijah channel	Oyster bed lagoon	Fort cay	
F_*ST*_	Cuero y Salado (n = 49)	–	**0.001**	**0.001**	**0.001**	
	Elijah channel (n = 11)	0.062	–	**0.003**	**0.016**	
	Oyster bed lagoon (n = 28)	0.027	0.034	–	**0.001**	
	Fort cay (n = 181)	0.105	0.051	0.071	–	

(c) Fort cay as two sites		Cuero y Salado	Elijah channel	Oyster bed lagoon	Fort cay K1	Fort cay K2
F_*ST*_	Cuero y Salado (n = 49)	–	**0.0001**	**0.0001**	**0.0001**	**0.0001**
	Elijah channel (n = 11)	0.062	–	**0.004**	**0.005**	**0.0001**
	Oyster bed lagoon (n = 28)	0.027	0.034	–	**0.005**	**0.0001**
	Fort cay K1 (n = 120)	0.043	0.032	0.014	–	**0.0001**
	Fort cay K2 (n = 61)	0.414	0.326	0.374	0.355	–

Significance values are in bold.

F_*ST*_ values are in the lower quartile and significance values (using AMOVA tests) in the upper quartile of each table.

Number of samples per site on which the analyses were undertaken (n) are in parentheses after the site name.

**Figure 3:**
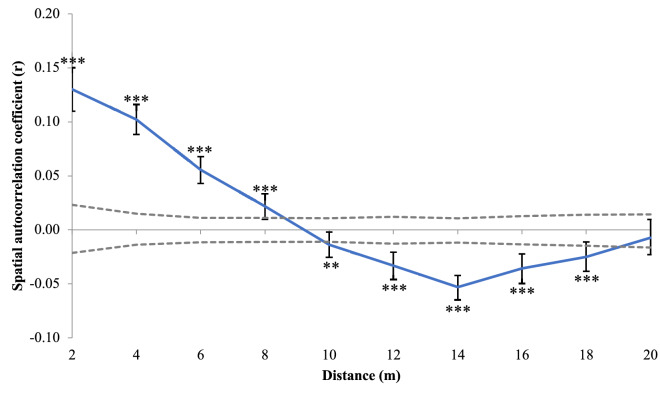
Spatial autocorrelation at 2 m spatial distance classes within the 400 m^2^ parcel of forest on Fort Cay. Gray dashed lines denote the upper and lower null confidence interval. Asterisk denotes significant correlations.

Genetic spatial autocorrelation analysis identified significant positive autocorrelation that progressively declined in intensity for the first four distance classes (2–8 m), followed by significant negative autocorrelation at the next five distance classes (10–18 m) (Fig. 3). No significant genetic spatial autocorrelation was observed at the last distance class of 20 m (Fig. 3).

### Large-scale genetic spatial structure

Pairwise F_*ST*_ analyses of the four sampling sites separated by distances of 6–115 km identified limited genetic structure among the four sites, with F_*ST*_ values ranging from 0.027 to 0.105 (Fig. 1b; Table 1b). Subsequent pairwise F_*ST*_ analyses of all sites, with individuals from Fort Cay divided into two clusters, K1 and K2, revealed individuals collected along the seaward fringes were more genetically similar to the other three sampled sites (F_*ST*_ = 0.014–0.062) (Fig. 1c; Table 1c). Greatest genetic structure was observed between mangroves from the interior of the parcel, assigned to cluster FC-K2, and all other mangroves from along the seaward fringe, i.e., CS, EC, OB, FC-K1 (F_*ST*_ = 0.326–0.414) (Fig. 1c; Table 1c).

## Discussion

Significant genetic structure was observed within the 400m^2^ parcel of *Rhizophora mangle* forest on Fort Cay, Honduras, with mangroves assigned to one of two clusters and significant genetic spatial autocorrelation observed across the parcel at 2 m spatial scales. Trees assigned to cluster FC-K1 were primarily associated with the seaward fringe, whereas individuals assigned to FC-K2 were located more in the interior of the parcel. Limited genetic structure was observed across the four mangrove stands separated by 6–115 km in the Honduran Caribbean. However, subsequent analyses, with mangroves from Fort Cay grouped by their K-means clustering assignment, demonstrated that this pattern was caused primarily by the relationship among individuals situated along the seaward fringe and individuals at the other sites. Much greater genetic differentiation was observed between mangroves located in the interior of Fort Cay and the other sites. This suggests high levels of connectivity between mangroves along shorelines, which has been previously observed^24^, but that the hypothesis of low or limited genetic structure at fine-scales within *R. mangle* forests should be rejected. The significant genetic differentiation observed among individuals assigned to cluster FC-K2 on Fort Cay compared to all other sites, suggests at least two recruitment events from distinct sources have occurred within the Fort Cay parcel.

### Fine-scale genetic structure

Our analysis of a small parcel of mangrove forest revealed significant genetic structure at fine spatial scales, with two genetically distinct clusters of trees identified in STRUCTURE and assigned through K-means clustering. Our findings provide evidence that refute the hypothesis that (*R. mangle*) mangrove forests lack genetic structure^13^, with significant genetic differences occurring at 8–10 m scales. We observed significant positive spatial autocorrelation at 2 m spatial scales, at 2–8 m distance classes, and significant negative genetic spatial autocorrelation at 10–18 m distance classes. These results indicate that individual clusters have a spatial dominance of 2–8 m, and trees outside of this boundary are significantly different and potentially from different sources, either from distinct parental families within the same forest or from spatially distant sources.

We suggest that the genetic structure observed, both in genetic spatial autocorrelation and pairwise *F*_ST_ analyses, could be the result of at least two different propagule sources which have recruited into this section of forest. The mangrove fringe, where wind and wave abatement primarily occurs, is the most dynamic area during storm events, and the part of the forest that suffers the most damage^28^ and provides protection to mangroves deeper within these systems. When the fringe is damaged by storms, there is the potential for recruitment of propagules from both local and distant sources, as storm waters are important drivers of mangrove dispersal and can facilitate long distance dispersal and range expansion of mangroves^29^. A total of 52 tropical storm and hurricane events have passed within 50 nautical miles of Fort Cay, from 1852 to 2016 (up to the time of sampling)^30^, the most severe was hurricane Mitch in 1998, which caused various levels of damage to mangrove stands throughout the Bay Islands, Honduras^31^. Such events could have had a role in shaping the genetic structure observed in the parcel of mangrove on Fort Cay. Damage to the fringe from tropical storms or hurricanes could have opened space for the recruitment of propagules from other populations that were dispersed by the storm event to the parcel. However, dispersal alone does not equate to recruitment success, as recruitment is influenced by numerous biotic and abiotic factors, including propagule predation rates^32^, sediment conditions^33^, and the presence of canopy gaps^28,34^. The sources and the mechanism of these different recruitment events is unknown, but with trees of various sizes associated with each of the identified clusters present, we suggest recruitment has persisted over various temporal and spatial scales. Whilst defining the drivers and spatial and temporal scales of dispersal and recruitment events are beyond the scope of this study, our results identify the influence of potential recruitment events, from either proximal or distal sources, on genetic diversity and structure at fine scales.

### Large-scale genetic structure

Limited genetic structure was observed in *R. mangle* forests between the four sampling sites within the Honduran Caribbean, at spatial scales of 6–115 km. These results suggest high levels of connectivity among mangroves within the Honduran Caribbean, and mirror findings from previous studies on both *R. mangle* and *Avicennia germinans* (black mangrove) in the Mesoamerican Reef region and Gulf of Mexico, at scales of 100’s km^35^. A more recent study observed greater genetic structure between *R. mangle* stands located between these two ecoregions, but, limited genetic structure within an ecoregion, at spatial scales of 10’s to 100’s km^19^. By placing the two clusters identified in Fort Cay in a wider context of *R. mangle* forests from across the Honduran Caribbean, we suggest that the connectivity observed at large-scales in many studies is potentially an artifact of sampling design. Mangroves assigned to cluster FC-K1 were predominantly associated with the seaward fringe of the parcel, and were most genetically similar to the three other sampling sites, where samples had also been collected from the seaward fringe. In contrast, individuals assigned to cluster FC-K2 were associated with the greatest levels of genetic differentiation to *R. mangle* trees sampled from the seaward fringe, including FC-K1. The limited genetic structure at the larger scale may be explained by propagules being dispersed by prevailing currents and recruiting along the seaward fringe, as propagules are capable of dispersing 1000’s km^1,36^. Similarly, larval models have demonstrated high levels of connectivity for other marine species in the region^37^. Alternatively, individuals assigned to FC-K1 may represent local recruitment and individuals of FC-K2 represent recruitment from another unknown population. Due to the higher frequency of individuals assigned to cluster FC-K1, local recruitment may be dominant, but interspersed with recruitment events from other more distant populations, and that layers of these events build up as the forest matures. We can only hypothesize about the drivers of the genetic structure observed, however, we can state unequivocally that sampling only the seaward fringe of a mangrove stand does not provide a complete overview of genetic structure of mangroves at larger spatial scales, and may greatly influence our interpretation of genetic connectivity within these ecosystems.

### Management and restoration implications

Genetic structure has implications for ecosystem function and the composition of associated biodiversity^17,38,39^. Maintaining genetic diversity within mangroves (and other ecosystems) is of increasing importance during an era of unprecedented climate change, and therefore population genetics should be an essential component of management and restoration frameworks^27,40,41^. To improve management and conservation efforts it is essential to have a greater understanding of the genetic structure of mangroves at the forest and seascape scale, and to ensure that management occurs at the appropriate spatial scales. Previous population genetic studies have increased our understanding of dispersal mechanisms and connectivity of mangrove populations (see Van der Stocken et al.^42^), and sampling scales of 5–30 m^18,19,43,44^, and up to 100 m^45^ have prevented sampling the same individual multiple times. However, most of these studies also only sampled along the seaward fringe of the mangrove. Our findings suggest that such sampling ranges are adequate, but based on the genetic structure observed here (i.e., positive spatial autocorrelation up to 8 m) we recommend sampling at similar spatial scales, 10–30 m, into the interior of mangrove forests. Sampling inland, not just along, a coastline will provide greater representation of the genetic structure within a population. This in turn can inform the spatial scale required for effective management of mangroves across seascapes, and can be used to monitor the effectiveness of restoration projects in maintaining genetic diversity, which are fundamental to the success of forest management^40,41^, and restoration frameworks (see Mijangos et al.^27^). Maintaining genetic diversity and gene flow of restored forests is a critical step in building resistance, resilience and adaptation of restored mangroves to future environmental conditions^46^. Understanding the genetic structure of mangroves at the forest scale can also help inform propagule source selection and planting practices, where required. This study provides further evidence of how genetic and genomic tools can improve the monitoring and evaluation of the management and restoration of mangroves^47,48^.

## Materials and methods

### Sample collection and DNA extraction

**Figure 4:**
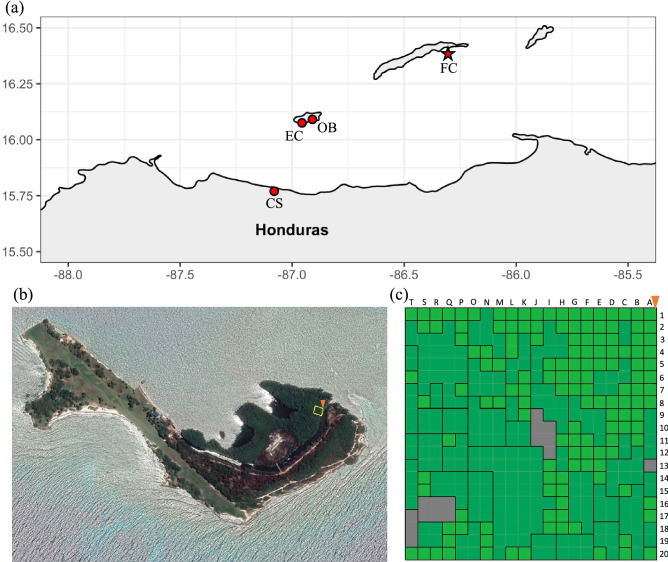
Map of **(a)** the Honduran north shore, **(b)** Fort Cay and the fine-scale sampling area, marked by the yellow square with the orange peg indicating the initial sampling square A1. **(c)** Schematic of the 400 m^2^ sampling grid and the position of the 181 Rhizophora mangle trees within the parcel. Black outlines demark individual trees; Light green—trees dominant within a 1 m^2^ area; Dark green—trees dominant in 2 m^2^ or greater; Gray represents no tree present (A13) or samples collected that did not sequence (I10, Q16, T17). Maps were created with R Studio^56^ using satellite images provided by Google Maps.

For the fine scale study, a 400 m^2^ area of mature mangrove forest comprising of only *R. mangle* was identified on Fort Cay, which is separated from the island of Roatan, Honduras, by a minimum of 1 km. The parcel was delineated using a 20 × 20 m sampling grid, and individual 1 × 1 m sampling cells were constructed in the forest using measuring tapes and ropes (sampling cell A1: N 16.404, W −86.282; Fig. 4). Sampling at Fort Cay (FC) was conducted over a three-day period between 24th and 26th April 2016. Two to three leaves were collected from the dominant tree within each of the sampling cells. Dominant trees were characterized as either the tree with the largest trunk within the sampling cell, or with the greatest canopy cover of the cell. Samples were collected for each cell, however, where a tree dominated two or more cells only one sample, from the first cell occupied by the tree, was used in the analysis.

Three further sites were also sampled for this study. Two of these were on the island of Utila; Oyster bed lagoon (OB) and Elijah canal (EC), and collections at these occurred on the 22nd and 23rd November 2016, respectively. The third site was on the Honduran mainland at Cuero y Salado (CS), where collection occurred on the 25th July 2017 (Fig. 4). Sampling of mangroves at these three sites was conducted along the seaward fringe, with care taken not to sample the same mangrove twice. Collections were conducted under research permit: DE-MP-001–2016 from the Instituto Nacional de Conservación y Desarrollo Forestal (ICF), Honduras. Our study complies with relevant institutional, national, and international guidelines and legislation.

At all sites, young leaves were preferentially sampled, due to lower concentrations of polyphenolics and other secondary metabolites compared to older leaves^49^, as secondary metabolites can cause shearing of DNA during extraction^50^. Upon collection, petioles were removed and leaves were broken in half to facilitate desiccation, and stored in individually labeled bags containing a 0.06–0.80 mm granular mix silica gel with cobalt indicator, prior to DNA extraction.

Prior to extraction approximately 1 cm^2^ of leaf tissue sample was placed in individually labeled 2.0 ml microcentrifuge tubes, and bathed in 500 µl of 100% ethanol for 3–4 days and then dried under fume hood for an additional two days. This process aided in the removal of secondary metabolites within leaves, and provided an additional dehydration step. Once dry, a single 5 mm stainless steel ball-bearing (Qiagen, Venlo, Netherlands) was added to each tube and samples were lysed using a Retsch (Düsseldorf, Germany) MM440 mixer mill (also known as a Tissuelyser). Samples were placed in one of two Tissuelyser adapter sets and lysed at 30 Hz for 1 min. Samples were flipped and swapped between Tissuelyser arms and lysed at 30 Hz for a further 1 min to facilitate consistent lysing across samples. DNA extraction from lysed tissue was conducted using the DNeasy 96 Plant Kit (Qiagen) following the manufacturers’ protocol.

### 2b-RAD-seq library preparation

2b-RAD-seq libraries were prepared using a modified version of the Wang et al.^51^ restriction site associated DNA (RAD) protocol. This methodology utilizes type IIB restriction enzymes that cut both upstream and downstream of the enzyme’s target site, resulting in the production of RAD tags of uniform length. Briefly, approximately 50–100 ng of high-quality genomic DNA (thin bright band on gel, with no smearing) from each sample was digested with the enzyme BcgI (New England BioLabs, Ipswich, USA), producing uniform 36 bp length fragments with random overhangs. Genomic digests were then ligated to a pair of partially double-stranded adaptors targeting a reduced subset of BcgI sites through a different reduction scheme depending on organism genome size. RAD tags were then amplified with sample-specific 5.6 bp or 6.6 bp dual-barcodes and Illumina adaptors. PCR products were visualized on a 2.0% agarose gel to verify the presence of the expected 160–170 bp target band (i.e., fragment, barcodes and adaptors included). Gel purification of the target band was carried out following protocols outlined in Guo et al.^52^. Amplification products were pooled at equimolar concentrations and sequenced on an Illumina HiSeq 3000 (San Diego, USA), at the Center for Genome Research and Biocomputing at Oregon State University, with single end sequence read lengths of 50 bp.

### SNP calling and quality control

Raw reads were downloaded from the Oregon State University online portal. Libraries for three trees failed to amplify. Successfully amplified libraries from the remaining 269 trees were processed using ipyrad v0.9.12^53^ on the Smithsonian Institution High Performance Computing Cluster (https://doi.org/10.25572/SIHPC). The genome of *R. apiculata*^54^, a close relative of *R. mangl*e, was used as the reference genome. In ipyrad v0.9.12^53^, all parameters were set to default, except for the following: data type = 2brad; restriction overhang = ‘TGCAG’; cluster threshold = 0.85; maximum barcodes mismatch = 0; filter adapters = 2; filter minimum trim length = 20; maximum alleles consent = 2; minimum samples per locus = 4; and, trim read = 0; and trim loci = 0. An initial panel of 113,626 loci was generated. We removed markers with 50% or more missing data and those with minor alleles frequencies of < 0.01^55^. Screening for null alleles, deviation from Hardy–Weinberg equilibrium and linkage disequilibrium were conducted in R Studio^56^ using the packages adegenet^57^, poppr^58^, and genepop^59^. Whilst the removal of markers due to Hardy–Weinberg disequilibrium may reduce the resolving power of the SNP data set, due to the Wahlund effect^60^, less than 30 SNPs were identified as deviating from Hardy–Weinberg equilibrium and were removed from the SNP panel. After post filtering and quality control, a panel of 677 informative SNPs was identified. The screening process was highly conservative, this was to reduce the effect of rare alleles being the underlying driver of structure within Fort Cay, the fine-scale analysis site.

### Statistical analyses

Population structure of *R. mangle* stands was analyzed using the software STRUCTURE v2.3.4^61,62^, with a burn-in length of 100,000, and Markov chain Monte Carlo replications set at 100,000. The model was run with K values of 1 through 10, with 10 permutations per K value. Optimal K values were selected from the highest delta K value generated by STRUCTURE HARVESTER^63^. This was conducted firstly for Fort Cay only, and subsequently for the four sites (CS, EC, OB and FC), and then for five sites, where individuals from Fort Cay were included as one of two sites, FC-K1 or FC-K2, based on their assigned cluster in the Fort Cay only analysis. Further analysis of individuals from Fort Cay to identify nested patterns of genetic structure, or hierarchical structure analysis, were conducted as recommended by Pritchard and Wen^64^ and Vähä et al.^65^. K-Means clustering analyses were conducted in GenoDive using the settings: cluster = individuals; method = Amova; run from 1 to 20 clusters. Convergence type was simulated annealing using 50,000 steps with 20 algorithm repeats, and best clustering according to Calinski & Harabasz’ pseudo-F^66^. Iterations of these analyses were conducted using “Bayesian Information Criterion (BIC)”, “Aikaike’s Information Criterion (AIC)” and “within-groups sum of squares” statistics. Pairwise F_*ST*_ analyses, using 50,000 permutations, were conducted in GenoDive^67^, on clusters K1 and K2 from Fort Cay, all four sampling sites, and five sites where mangroves from Fort Cay were included as one of two sites, FC-K1 and FC-K2.

Spatial autocorrelation analysis between pairs of individuals at specified distance classes were performed to assess fine-scale genetic structure of mangroves at Fort Cay. In GenAlEx 6.5^68^, we calculated pairwise inter-individual genetic distances as outlined in Smouse and Peakall^69^ and geographic distances measured between the grid locations of individual trees. Consistent with our systematic sampling design, we performed the spatial autocorrelation analysis with even distance classes (10 total classes, each encompassing 2 m intervals) which maintained large numbers of sample pairs (565–2204) at each distance class. An autocorrelation coefficient (*r*) was calculated for each distance class and plotted as a spatial genetic correlogram. Null 95% confidence intervals at each distance class were generated via 999 random permutations of all samples and 95% confidence intervals around each *r* value were generated via 10^3^ bootstrap replicates of the samples at the respective distance class. As described in Peakall et al.^70^, we accepted statistical significance of spatial autocorrelation at a distance class when (1) the *r* value exceeded the null confidence interval and (2) the confidence interval around the *r* value did not overlap with zero.

## Data Availability

The annotated SNPs are available on Figshare: DOI: 10.25573/data.17898698.
